# Joint Smoothed *l*_0_-Norm DOA Estimation Algorithm for Multiple Measurement Vectors in MIMO Radar

**DOI:** 10.3390/s17051068

**Published:** 2017-05-08

**Authors:** Jing Liu, Weidong Zhou, Filbert H. Juwono

**Affiliations:** 1 College of Automation, Harbin Engineering University, Harbin 150001, China; liujing@hrbeu.edu.cn; 2 School of Electrical, Electronic and Computer Engineering, The University of Western Australia, Perth 6009, Australia; 3 Department of Electrical Engineering, Universitas Indonesia, Depok 16424, Indonesia; filbert@ieee.org

**Keywords:** direction-of-arrival estimation, joint smoothed *l*_0_-norm, multiple measurement vectors, sparse signal reconstruction, MIMO radar

## Abstract

Direction-of-arrival (DOA) estimation is usually confronted with a multiple measurement vector (MMV) case. In this paper, a novel fast sparse DOA estimation algorithm, named the joint smoothed $l_{0}$-norm algorithm, is proposed for multiple measurement vectors in multiple-input multiple-output (MIMO) radar. To eliminate the white or colored Gaussian noises, the new method first obtains a low-complexity high-order cumulants based data matrix. Then, the proposed algorithm designs a joint smoothed function tailored for the MMV case, based on which joint smoothed $l_{0}$-norm sparse representation framework is constructed. Finally, for the MMV-based joint smoothed function, the corresponding gradient-based sparse signal reconstruction is designed, thus the DOA estimation can be achieved. The proposed method is a fast sparse representation algorithm, which can solve the MMV problem and perform well for both white and colored Gaussian noises. The proposed joint algorithm is about two orders of magnitude faster than the $l_{1}$-norm minimization based methods, such as $l_{1}$-SVD (singular value decomposition), RV (real-valued) $l_{1}$-SVD and RV $l_{1}$-SRACV (sparse representation array covariance vectors), and achieves better DOA estimation performance.

## 1. Introduction

Colocated multiple-input multiple-output (MIMO) radar has attracted a growing interest recently because it can achieve higher resolution and better parameter identification compared with conventional phased-array radar [1]. As one type of colocated MIMO radar, the monostatic MIMO radar is equipped with closely-located transmit and receive arrays, which result in the same angle for direction-of-departure (DOD) and direction-of-arrival (DOA) [2]. Aiming at DOA estimation, a large quantity of methods have been proposed, most of them are based on the signal and noise subspaces [3,4,5,6], such as the representative MUSIC (multiple signal classification) algorithm [3] and ESPRIT (estimation of signal parameters via rotational invariance techniques) algorithm [4].

In the area of sensor array signal processing, sparse representation is an important technique to estimate the parameters. When reconstructing the sparse signal, to avoid the NP-hard (non-deterministic polynomial-time hard) $l_{0}$-norm minimization, different methods such as those based on the relaxed constraint $l_{1}$-norm minimization [7], the focal underdetermined system solution (FOCUSS) [8] and the sparse Bayesian learning (SBL) [9] have been proposed. These algorithms were originally designed for the single measurement vector (SMV) problem. Then, to extend them to the multiple measurement vector (MMV) case, some algorithms, e.g., M-FOCUSS and M-SBL, were proposed [10,11]. Recently, the emerging application that exploits the sparse representation technique to achieve the DOA estimation has been increasingly attractive. Compared with the alternative methods, sparse representation-based DOA estimation methods can achieve higher angle resolution and better adapt to some challenging scenarios such as low signal-to-noise ratio (SNR), as discussed in [7]. Among the abovementioned sparse signal reconstruction methods, $l_{1}$-norm minimization is widely used for sparse DOA estimation because of its desirable solution accuracy and reasonable computing speed [7,10,12]. The DOA estimation algorithms using $l_{1}$-norm minimization include $l_{1}$-SRACV (sparse representation array covariance vectors) [13], real-valued (RV) $l_{1}$-SRACV [14], and the iterative algorithm with reweighting $l_{1}$-norm minimization [15]. All of these methods involve the SMV problem when recovering the signal. Aiming at the MMV case, the $l_{1}$-SVD (singular value decomposition) [7], RV $l_{1}$-SVD [16], reweighted $l_{1}$-SVD [17] and CMSR (covariance matrix sparse representation) [10] were proposed for DOA estimation. However, the computational complexity of the $l_{1}$-norm minimization is relatively large compared with that of the fast sparse representation method named smoothed $l_{0}$-norm algorithm, which was proposed in [18,19] under the SMV circumstance, and applied in [20,21] for the imaging and the power-line communications. To achieve fast sparse DOA estimation, by designing a reweighted continuous function in the SMV case, the reweighted smoothed $l_{0}$-norm (RSL0) algorithm was proposed in [22] based on the covariance vector. With enlarged array aperture and better angle estimation performance, the RSL0 algorithm [22] is about two orders of magnitude faster than the $l_{1}$-norm minimization based methods.

The previously mentioned DOA estimation methods are all proposed under the ideal circumstance with Gaussian white noise. However, the noise is often correlated in practical, thus the additive noise is spatially modeled as Gaussian colored noise rather than white noise. It has been verified that, with colored noise, the performance of the conventional algorithms including the methods mentioned above, is seriously degraded, except for the specially designed methods such as those based on fourth-order cumulants (FOC) [12,23,24]. These FOC-based methods are computationally expensive when they are applied to estimate the DOAs for MIMO radar. In [25], the reduced-dimension (RD) FOC-based sparse representation method (RD $l_{1}$-SRFOC) was proposed for DOA estimation in MIMO radar with array errors. RD $l_{1}$-SRFOC is an $l_{1}$-norm minimization based sparse representation method, which uses the high-order cumulants to deal with the colored noise and simultaneously solves the problem of mutual coupling.

It can be concluded that the existing sparse DOA estimation should improve the following problems: (I) for the $l_{1}$-norm minimization based methods, the computation complexities need to be lowered down and the computing time needs speeding up; (II) the fast sparse DOA estimation algorithm, i.e., the reweighted smoothed $l_{0}$-norm algorithm [22], is based on the covariance vector that is deemed the SMV problem. The fast sparse algorithm tailored for the MMV case needs to be studied and put forward; and (III) fast sparse algorithms for solving the colored Gaussian noise have not been available so far.

To solve the three problems stated above, a joint smoothed $l_{0}$-norm algorithm for DOA estimation in the MIMO radar is proposed in this paper. The new sparse algorithm first obtains a low-complexity FOC-based data matrix to eliminate the white or colored Gaussian noises. Secondly, the proposed algorithm designs a joint smoothed function tailored for the MMV case. Thirdly, with the MMV-based joint smoothed function, a joint smoothed $l_{0}$-norm sparse representation framework is constructed. Finally, the corresponding gradient-based sparse signal reconstruction is designed, and then DOA estimation is achieved. The proposed algorithm is a fast sparse DOA estimation algorithm, which can solve the multiple measurement vector problem, and perform well for both of the white and colored Gaussian noise environments. The proposed algorithm is about two orders of magnitude faster than the $l_{1}$-norm minimization based methods. This is because it approximates the $l_{0}$-norm by a joint smoothed function and performs the gradient-based steepest ascent scheme, which avoids the convex optimization problem involved in the $l_{1}$-norm minimization. The proposed algorithm can provide better DOA estimation performance than $l_{1}$-SVD, RV $l_{1}$-SVD and RV $l_{1}$-SRACV methods.

The rest of this paper is organized as follows. Section 2 presents the MIMO radar system and array signal models. In Section 3, the implementation process of the proposed algorithm is described in detail. Section 4 gives some related remarks and discussions regarding the advantages, the extended applications, the computational complexity and the noise elimination of the proposed algorithm. The experimental results of different methods are compared in Section 5. Finally, Section 6 concludes this paper.

Throughout the paper, the notations ${\left(\cdot \right)}^{*}$, ${\left(\cdot \right)}^{T}$, ${\left(\cdot \right)}^{H}$, ${\left(\cdot \right)}^{-1}$ and ${\left(\cdot \right)}^{+}$ denote conjugation, transpose, conjugate transpose, inverse and pseudo-inverse, respectively. $I_{K}$ represents a K×K-dimensional unit matrix. $\left|\right|\cdot {\left|\right|}_{0}$ and $\left|\right|\cdot {\left|\right|}_{2}$ denote the $l_{0}$-norm and the $l_{2}$-norm, respectively. In addition, we use ⊗, E(·) and vec(·) to separately indicate the Kronecker product, the expectation and the vectorization operators.

## 2. Problem Formulation

**Figure 1:**
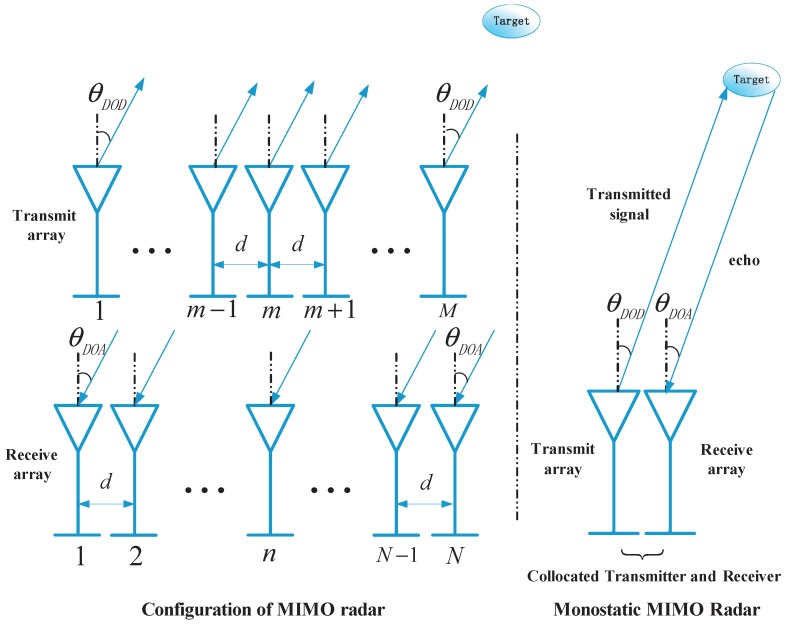
Configuration of monostatic multiple-input multiple-output radar.

A narrowband monostatic MIMO radar system is considered, and it is shown in Figure 1. The transmit and the receive arrays of this system are equipped with *M* transmit and *N* receive antennas, respectively. The arrays are both half-wavelength spaced uniform linear arrays (ULAs), whose effects of the array errors, including mutual coupling and gain-phase errors, can be ignored. The transmitting antennas transmit *M* orthogonal narrowband waveforms, such as BPSK (binary phase shift keying) modulated signal waveforms. In the far field, there are *P* uncorrelated targets regarded as point scatterers at the same range. In addition, it is assumed that P≤M+N−2 [22,25,26]. Thus, at the receive array, the *N* antennas are impinged by the echo signals reflected by the *P* targets. Because of the closely located arrays in the monostatic MIMO radar, for the *p*th target, DOA and DOD are the same and can be denoted by $\theta_{p}$. Then, by matched filtering operation, the N×1 dimensional complex envelop of the output of the *m*th carrier matched filter is expressed as [14,27]

(1) $$\begin{array}{c} x_{m}\left(t\right)=\sum_{p=1}^{P}a_{r}\left(\theta_{p}\right)a_{tm}^{T}\left(\theta_{p}\right)s_{p}\left(t\right)+n_{m}\left(t\right), \end{array}$$

where $a_{r}\left(\theta_{p}\right)={\left[1,e^{j\pi \sin(\theta_{p})},e^{j\pi 2\sin(\theta_{p})},\ldots ,e^{j\pi \left(N-1\right)\sin\left(\theta_{p}\right)}\right]}^{T}$ is the receive steering vector, $a_{tm}$ is the *m*th element of the transmit steering vector $a_{t}\left(\theta_{p}\right)={\left[1,e^{j\pi \sin(\theta_{p})},e^{j\pi 2\sin(\theta_{p})},\ldots ,e^{j\pi \left(M-1\right)\sin\left(\theta_{p}\right)}\right]}^{T}$, $s_{p}\left(t\right)$ contains the target reflection coefficient and the transmitted baseband signal such as non-circular signal, and $n_{m}\left(t\right)$ is the noise vector after the *m*th matched filter. After all the matched filters, the received data vector, i.e., the vector composed of the outputs of the *M* matched filters, is given by [2,25,28]

(2) $$\begin{array}{cc} x(t) & =\mathrm{vec}({\left[x_{1}^{T}\left(t\right),\ldots ,x_{M}^{T}\left(t\right)\right]}^{T})\\ & =As(t)+n(t), \end{array}$$

where $x\left(t\right)\in C^{MN\times 1}$ and $n\left(t\right)={\left[n_{1}^{T}\left(t\right),\ldots ,n_{M}^{T}\left(t\right)\right]}^{T}\in C^{MN\times 1}$ is the zero-mean Gaussian white or colored noise vector. $s\left(t\right)={\left[s_{1}\left(t\right),s_{2}\left(t\right),\ldots ,s_{P}\left(t\right)\right]}^{T}\in C^{P\times 1}$ is the reflected signal vector after the *M* carrier matched filters, and it is assumed to be statistically independent and non-Gaussian with zero-mean. The target model is considered as the classical Swerling case II, namely, the radar cross section (RCS) fluctuations are constant during a snapshot period and vary independently from snapshot to snapshot [29]. n(t) and s(t) are independent of each other. Moreover, $A\in C^{MN\times P}$ is the transmit–receive steering matrix, and its detailed expression is

(3) $$\begin{array}{cc} A & =[a_{t}\left(\theta_{1}\right)⊗a_{r}\left(\theta_{1}\right),\ldots ,a_{t}\left(\theta_{P}\right)⊗a_{r}\left(\theta_{P}\right)]\\ & =[a\left(\theta_{1}\right),\ldots ,a\left(\theta_{P}\right)], \end{array}$$

where $a\left(\theta_{p}\right)=a_{t}\left(\theta_{p}\right)⊗a_{r}\left(\theta_{p}\right)\in C^{MN\times 1}$ is the transmit–receive steering vector for p=1,2,…,P. Therefore, by collecting *J* snapshots, the MN×J dimensional received data matrix in monostatic MIMO radar is represented as

(4) X=AS+N,

where $S=\left[s\left(t_{1}\right),\ldots ,s\left(t_{J}\right)\right]\in C^{P\times J}$ and $N=\left[n\left(t_{1}\right),\ldots ,n\left(t_{J}\right)\right]\in C^{MN\times J}$ are the signal matrix and complex Gaussian noise matrix, respectively. Based on the received data X, sparse DOA estimation can be viewed as the signal reconstruction that subjects to [14]

(5) $$X=A_{\hat{\theta }}S_{\hat{\theta }}+N,$$

where $A_{\hat{\theta }}=\left[a\left({\hat{\theta }}_{1}\right),\ldots ,a\left({\hat{\theta }}_{L}\right)\right]$ is the complete dictionary with the discrete sample grid ${\left\{{\hat{\theta }}_{i}\right\}}_{i=1}^{L}$, L≫P. $S_{\hat{\theta }}$ has the same row support with S. Let $s_{\hat{\theta }}=\left[s\left({\hat{\theta }}_{1}\right),\ldots ,s\left({\hat{\theta }}_{L}\right)\right]$ be a sparse vector whose *i*th element can be equal to the $l_{2}$-norm of the *i*th row in $S_{\hat{\theta }}$. To obtain the sparsest solution of Equation (5), an ideal constraint is the $l_{0}$-norm method by minimizing the nonzero number of $s_{\hat{\theta }}$, which can be expressed as

(6) $$\min∥s_{\hat{\theta }}{∥}_{0},\;s.t.\;X=A_{\hat{\theta }}S_{\hat{\theta }}+N.$$

Unfortunately, the $l_{0}$-norm minimization method in Equation (6) is NP-hard. To solve this problem, conventional sparse DOA estimation methods [7,10,13,14,15,17] can obtain the DOAs with the $l_{1}$-norm minimization. However, the computational complexity of the $l_{1}$-norm minimization is relatively large. In [22], by solving the array covariance vector based SMV sparse signal reconstruction problem, the reweighted smoothed $l_{0}$-norm algorithm greatly improves the computation speed. To develop a fast sparse DOA estimation algorithm tailored for the MMV problem existed in the MIMO radar systems, a joint smoothed $l_{0}$-norm algorithm is proposed in the following.

## 3. The Proposed Algorithm

### 3.1. High-Order Cumulants Based Data Matrix Construction

Based on Equation (3), the detailed expression of the steering vector $a(\theta_{p})$ is

(7) $$\begin{array}{cc} a(\theta_{p}) & ={\left[a_{r}^{T}\left(\theta_{p}\right),e^{j\pi \sin(\theta_{p})}a_{r}^{T}\left(\theta_{p}\right),e^{j\pi \left(M-1\right)\sin\left(\theta_{p}\right)}a_{r}^{T}\left(\theta_{p}\right)\right]}^{T}\\ & ={\left[1,\ldots ,z^{N-1},z,\ldots ,z^{N},\ldots ,z^{M-1},\ldots ,z^{M+N-2}\right]}^{T}, \end{array}$$

with $z=e^{j\pi \sin(\theta_{p})}$. It can be observed that there are only M+N−1 distinct elements in $a(\theta_{p})$; thus, the dimension of the received data vector x(t) can be reduced. Let $b(\theta_{p})$ be a new steering vector composed of the distinct elements. It is written as

(8) $$b\left(\theta_{p}\right)={\left[1,e^{j\pi \sin(\theta_{p})},e^{j\pi 2\sin(\theta_{p})},\ldots ,e^{j\pi \left(M+N-2\right)\sin\left(\theta_{p}\right)}\right]}^{T},$$

where $b\left(\theta_{p}\right)\in C^{(M+N-1)\times 1}$. According to the element structures of $a(\theta_{p})$ and $b(\theta_{p})$, their relationship can be expressed as [25]

(9) $$a\left(\theta_{p}\right)=\mathrm{Gb}\left(\theta_{p}\right),$$

where $G={\left[L_{0}^{T},L_{1}^{T},\cdots ,L_{M-1}^{T}\right]}^{T}\in C^{MN\times (M+N-1)}$ and $L_{m}=\left[0_{N\times m},I_{N},0_{N\times (M-m-1)}\right]\in C^{N\times (M+N-1)}$ for m=0,1,...,M−1. Based on the relationship in Equation (9), a reduced-dimensional matrix can be defined as [25]

(10) $$R={\left(G^{H}G\right)}^{\left(-\frac{1}{2}\right)}G^{H}\in C^{\left(M+N-1\right)\times MN}.$$

Therefore, the dimensional reduction for the received data is carried out as follows [25]:

(11) $$\begin{array}{cc} \tilde{x}\left(t\right) & =RGBs(t)+Rn(t)\\ & ={\left(G^{H}G\right)}^{\left(-\frac{1}{2}\right)}G^{H}GBs\left(t\right)+\tilde{n}\left(t\right)\\ & =FBs\left(t\right)+\tilde{n}\left(t\right), \end{array}$$

where $\tilde{x}\left(t\right)=Rx\left(t\right)\in C^{(M+N-1)\times 1}$, $B=\left[b\left(\theta_{1}\right),\ldots ,b\left(\theta_{P}\right)\right]\in C^{(M+N-1)\times P}$ and $\tilde{n}\left(t\right)\in C^{(M+N-1)\times 1}$ are the new low-dimensional received data vector, transmit–receive steering matrix and noise vector, respectively. In addition, $F={\left(G^{H}G\right)}^{\frac{1}{2}}$ is a known diagonal matrix, and its (i,i)th element can be expressed as

(12) $$\begin{array}{c} F\left(i,i\right)=\left\{\begin{array}{c} \sqrt{i}\;\;i=1,2,\ldots ,\beta \\ \sqrt{\beta }\;\;i=\beta +1,\beta +2,\ldots ,\beta +\tau -1\\ \sqrt{M+N-i}\;\;i=\beta +\tau ,\ldots ,M+N-1, \end{array}\right. \end{array}$$

with β=min(M,N) and τ=|M−N|+2. Note that the noise vector $\tilde{n}\left(t\right)$ remains Gaussian after the dimensional reduction, for asymptotic normal distribution has the invariance speciality of linear transformation. To eliminate the Gaussian white noise or colored noise in $\tilde{x}\left(t\right)$, fourth-order cumulant is exploited under the circumstance of collecting *J* snapshots. The definition of the FOC is given as follows [12,24,25]

(13) $$\begin{array}{cc} C_{4\tilde{x}}\left(k_{1},k_{2},k_{3},k_{4}\right) & =\mathrm{cum}\left\{{\tilde{x}}_{k_{1}},{\tilde{x}}_{k_{2}}^{*},{\tilde{x}}_{k_{3}},{\tilde{x}}_{k_{4}}^{*}\right\}=E\left({\tilde{x}}_{k_{1}}{\tilde{x}}_{k_{2}}^{*}{\tilde{x}}_{k_{3}}{\tilde{x}}_{k_{4}}^{*}\right)-E\left({\tilde{x}}_{k_{1}}{\tilde{x}}_{k_{2}}^{*}\right)E\left({\tilde{x}}_{k_{3}}{\tilde{x}}_{k_{4}}^{*}\right)\\ & -E\left({\tilde{x}}_{k_{1}}{\tilde{x}}_{k_{3}}\right)E\left({\tilde{x}}_{k_{2}}^{*}{\tilde{x}}_{k_{4}}^{*}\right)-E\left({\tilde{x}}_{k_{1}}{\tilde{x}}_{k_{4}}^{*}\right)E\left({\tilde{x}}_{k_{2}}^{*}{\tilde{x}}_{k_{3}}\right), \end{array}$$

where $E\left({\tilde{x}}_{k_{1}}{\tilde{x}}_{k_{2}}^{*}{\tilde{x}}_{k_{3}}{\tilde{x}}_{k_{4}}^{*}\right)\approx \frac{1}{J}\sum_{t=1}^{J}{\tilde{x}}_{k_{1}}\left(t\right){\tilde{x}}_{k_{2}}^{*}\left(t\right){\tilde{x}}_{k_{3}}\left(t\right){\tilde{x}}_{k_{4}}^{*}\left(t\right)$, $E\left({\tilde{x}}_{k_{1}}{\tilde{x}}_{k_{2}}^{*}\right)\approx \frac{1}{J}\sum_{t=1}^{J}{\tilde{x}}_{k_{1}}\left(t\right){\tilde{x}}_{k_{2}}^{*}\left(t\right)$, ${\tilde{x}}_{k_{i}}$ stands for the $k_{i}$th element in $\tilde{x}$, and $1\leq k_{i}\leq M+N-1$ for i=1,2,3,4. With the assumptions about the noise in Equation (2), the fourth-order cumulant of Gaussian noise has the following property:

(14) $$C_{4\tilde{n}}\left(k_{1},k_{2},k_{3},k_{4}\right)=\mathrm{cum}\left\{{\tilde{n}}_{k_{1}},{\tilde{n}}_{k_{2}}^{*},{\tilde{n}}_{k_{3}},{\tilde{n}}_{k_{4}}^{*}\right\}=0,$$

where $C_{4\tilde{n}}\left(k_{1},k_{2},k_{3},k_{4}\right)$ represents the fourth-order cumulant of $\tilde{n}$ corresponding to the indices $\left(k_{1},k_{2},k_{3},k_{4}\right)$. For the signal assumed in Equation (2), the fourth-order cumulant satisfies

(15) $$\begin{array}{c} C_{4s}\left(p_{1},p_{2},p_{3},p_{4}\right)=\left\{\begin{array}{c} c_{p}\;\;p_{1}=p_{2}=p_{3}=p_{4}=p\\ 0\;\;\;otherwise, \end{array}\right. \end{array}$$

where $c_{p}=\mathrm{cum}\left\{s_{p},s_{p}^{*},s_{p},s_{p}^{*}\right\}$ is the FOC of the signal for the *p*th target, and $1\leq p_{i}\leq P$ for i=1,2,3,4. Based on Equations (14) and (15), an FOC-based data matrix can be constructed with its $\left(k_{1},k_{2}\right)$th element being obtained from

(16) $$\begin{array}{cc} Y(k_{1},k_{2}) & =C_{4\tilde{x}}\left(k_{1},k_{2},k_{2},k_{2}\right)\\ & =\mathrm{cum}\{{\tilde{x}}_{k_{1}},{\tilde{x}}_{k_{2}}^{*},{\tilde{x}}_{k_{2}},{\tilde{x}}_{k_{2}}^{*}\}\\ & =\sum_{p=1}^{P}{\left[Fb\left(\theta_{p}\right)\right]}_{k_{1}}{\left[Fb^{*}\left(\theta_{p}\right)\right]}_{k_{2}}{\left|F\left(k_{2},k_{2}\right)\right|}^{2}c_{p}, \end{array}$$

where ${\left[Fb\left(\theta_{p}\right)\right]}_{k_{1}}$ and ${\left[Fb^{*}\left(\theta_{p}\right)\right]}_{k_{2}}$ are the $k_{1}$th and the $k_{2}$th elements in $Fb(\theta_{p})$ and $Fb^{*}\left(\theta_{p}\right)$. As a result, the data matrix Y can be expressed as

(17) $$Y=FBD_{s}{\left(F^{3}B\right)}^{H},$$

where $D_{s}=\mathrm{diag}\left(c_{1},c_{2},\ldots ,c_{P}\right)$ is a diagonal matrix composed of the fourth-order cumulants for $s_{p}$, p=1,2,…,P. Then, the singular value decomposition of Y can be performed as follows:

(18) $$Y=U\Lambda V^{H},$$

where U is the left singular vector, V is the right singular vector, and $\Lambda =\mathrm{diag}(\lambda_{1},\lambda_{2},\ldots ,\lambda_{M+N-1})$ is the singular value matrix with $\lambda_{1}\geq \lambda_{2}\geq \ldots \geq \lambda_{M+N-1}$. Therefore, the signal subspace $V_{s}$ is obtained by extracting the first *P* right singular vectors that correspond to $\left(\lambda_{1},\lambda_{2},\ldots ,\lambda_{P}\right)$. In addition, the last M+N−1−P left singular vectors corresponding to $\left(\lambda_{P+1},\lambda_{P+2},\ldots ,\lambda_{M+N-1}\right)$ are used to make up the noise subspace $U_{n}\in C^{\left(M+N-1)\times (M+N-1-P\right)}$. With $V_{s}\in C^{(M+N-1)\times P}$, Y can be transformed into a dimension-reduced data matrix, that is

(19) $$\begin{array}{c} Y_{s}=YV_{s}=FBD_{s}{\left(F^{3}B\right)}^{H}V_{s}, \end{array}$$

where $Y_{s}\in C^{(M+N-1)\times P}$.

### 3.2. Designs of Joint Smoothed Function and Joint Smoothed $l_{0}$-Norm Framework for MMV Case

In the sparse representation framework for DOA estimation, a complete dictionary containing all interest DOAs is required. Thus, let ${\left\{{\hat{\theta }}_{i}\right\}}_{i=1}^{L}$ be the discrete sample grid. According to Equation (19), the complete dictionary can be constructed as $B_{\hat{\theta }}=\left[b\left({\hat{\theta }}_{1}\right),b\left({\hat{\theta }}_{2}\right),\ldots ,b\left({\hat{\theta }}_{L}\right)\right]\in C^{\left(M+N-1\right)\times L}$. The DOA estimation is considered as a sparse signal reconstruction problem that is subject to

(20) $$Y_{s}=FB_{\hat{\theta }}T_{\hat{\theta }},$$

where $T_{\hat{\theta }}\in C^{L\times P}$ is a sparse matrix and has the same row support with $T=D_{s}{\left(F^{3}B\right)}^{H}V_{s}$. Then, DOA estimation can be achieved by measuring the sparsity of $T_{\hat{\theta }}$. Note that, in the sparse signal recovery problem in Equation (20), the low-dimensional data $Y_{s}$ is a matrix rather than a vector. Therefore, recovering $T_{\hat{\theta }}$ involves the MMV problem. To develop a fast sparse DOA estimation algorithm that can solve the MMV problem, in the following, the proposed joint smoothed $l_{0}$-norm algorithm designs a new joint smoothed function and then derives its new steepest ascent scheme. For obtaining the smoothed estimation of the sparsity of $T_{\hat{\theta }}$, we first design a joint continuous function as follows

(21) $$f_{\sigma }\left[T_{\hat{\theta }}\left(i,:\right)\right]=\exp\left\{-\left[|T_{\hat{\theta }}\left(i,1\right){|}^{2}+|T_{\hat{\theta }}\left(i,2\right){|}^{2}+\ldots +|T_{\hat{\theta }}\left(i,P\right){|}^{2}\right]/2P\sigma^{2}\right\},$$

where $T_{\hat{\theta }}\left(i,p\right)$ is the (i,p)th element of $T_{\hat{\theta }}$ for p=1,2,…,P and i=1,2,…,L. Thus, the result of the designed joint continuous function $f_{\sigma }\left[T_{\hat{\theta }}\left(i,:\right)\right]$ can be expressed as follows:

(22) $$\lim_{\sigma \to 0}f_{\sigma }\left[T_{\hat{\theta }}\left(i,:\right)\right]=\left\{\begin{array}{c} 1\;\;T_{\hat{\theta }}^{l_{2}}\left(i\right)=0\\ 0,\;\;T_{\hat{\theta }}^{l_{2}}\left(i\right)\neq 0, \end{array}\right.$$

where $T_{\hat{\theta }}^{\left(l_{2}\right)}\in C^{L\times 1}$ is the L×1 dimensional vector, and $\left[T_{\hat{\theta }}^{\left(l_{2}\right)}\right]\left(i\right)=∥T_{\hat{\theta }}\left(i,:\right){∥}_{2}$. Namely, the *i*th element of $T_{\hat{\theta }}^{\left(l_{2}\right)}$ is the $l_{2}$-norm of the *i*th row in $T_{\hat{\theta }}$. Then, a series of weights $\left[r_{w1},r_{w2},\ldots ,r_{wL}\right]$ are introduced into the joint continuous function $f_{\sigma }\left[T_{\hat{\theta }}\left(i,:\right)\right]$. Therefore, in the proposed joint smoothed $l_{0}$-norm algorithm, the final design of the joint smoothed function is

(23) $$\begin{array}{cc} f_{w\sigma }\left[T_{\hat{\theta }}\left(i,:\right)\right] & =\exp\{-\frac{r_{wi}}{2P\sigma^{2}}\sum_{p=1}^{P}\left[|T_{\hat{\theta }}\left(i,p\right){|}^{2}\right]\}\\ & =e^{-\frac{r_{wi}}{2P\sigma^{2}}\sum_{p=1}^{P}\left[|T_{\hat{\theta }}\left(i,p\right){|}^{2}\right]}, \end{array}$$

where $r_{wi}$ is the weight coefficient, which is obtained from $r_{wi}=∥{\left[Fb\left({\hat{\theta }}_{i}\right)\right]}^{H}U_{n}{∥}_{2}/r_{max}$ with $r_{max}=\max\left\{∥{\left[Fb\left({\hat{\theta }}_{1}\right)\right]}^{H}U_{n}{∥}_{2},∥{\left[Fb\left({\hat{\theta }}_{2}\right)\right]}^{H}U_{n}{∥}_{2},\ldots ,∥{\left[Fb\left({\hat{\theta }}_{L}\right)\right]}^{H}U_{n}{∥}_{2}\right\}$. In Equation (23), the preset parameter σ is known and adjusts the smoothness of $f_{w\sigma }\left[T_{\hat{\theta }}\left(i,:\right)\right]$. In addition, *P* stands for the total number of the columns in the data matrix $Y_{s}$, namely, the column number of the sparse matrix $T_{\hat{\theta }}$. For the true target DOA $\theta_{p}$, $0<r_{wp}\ll 1$ because of the orthogonality between the signal subspace and the noise subspace [5]. Therefore, the approximate value of the joint smoothed function $f_{w\sigma }\left[T_{\hat{\theta }}\left(i,:\right)\right]$ corresponding to ${\hat{\theta }}_{i}$ can be calculated as follows:

(24) $$f_{w\sigma }\left[T_{\hat{\theta }}\left(i,:\right)\right]\approx \left\{\begin{array}{c} 1\;\;r_{wi}\to 1,T_{\hat{\theta }}^{l_{2}}\left(i\right)\ll \sigma \\ 0\;\;r_{wi}\ll 1,T_{\hat{\theta }}^{l_{2}}\left(i\right)\gg \sigma , \end{array}\right.$$

where a small σ is required to guarantee the approximation. Based on Equation (24), the sparsity of $T_{\hat{\theta }}$ can be measured by

(25) $$\begin{array}{c} \left|\right|T_{\hat{\theta }}^{l_{2}}{\left|\right|}_{0}\approx L-\sum_{i=1}^{L}f_{w\sigma }\left[T_{\hat{\theta }}\left(i,:\right)\right]=L-F_{w\sigma }\left(T_{\hat{\theta }}^{l_{2}}\right), \end{array}$$

where $F_{w\sigma }\left(T_{\hat{\theta }}^{l_{2}}\right)=\sum_{i=1}^{L}f_{w\sigma }\left[T_{\hat{\theta }}\left(i,:\right)\right]$. According to Equation (25), to recover the sparse matrix $T_{\hat{\theta }}$, the minimization of $\left|\right|T_{\hat{\theta }}^{l_{2}}{\left|\right|}_{0}$ can be turned into the maximization of $F_{w\sigma }\left(T_{\hat{\theta }}^{l_{2}}\right)$. As a consequence, the MMV-based joint smoothed $l_{0}$-norm sparse representation framework is represented as

(26) $$\max_{T_{\hat{\theta }}}F_{w\sigma }\left(T_{\hat{\theta }}^{l_{2}}\right),\;s.t.\;Y_{s}=FB_{\hat{\theta }}T_{\hat{\theta }}.$$

### 3.3. Design of the MMV-Based Signal Reconstruction in the Joint Smoothed $l_{0}$-Norm Algorithm

For the joint smoothed function $F_{w\sigma }\left(T_{\hat{\theta }}^{l_{2}}\right)$, the steepest ascent based on the gradient is designed to recover the sparse signal. Aiming at the sparse signal reconstruction in Equation (26) that involves the MMV problem, an initial matrix is needed for the joint smoothed $l_{0}$-norm algorithm to start the steepest ascent process. The initial matrix $U_{0}^{\hat{\theta }}$ can be chosen as the solution of Equation (26) with σ→∞, i.e., the minimum $l_{2}$-norm solution calculated by

(27) $$U_{0}^{\hat{\theta }}={\left(FB_{\hat{\theta }}\right)}^{+}Y_{s}\in C^{(M+N-1)\times P},$$

where ${\left(FB_{\hat{\theta }}\right)}^{+}$ is the pseudo-inverse of $FB_{\hat{\theta }}$. When recovering the sparse signal for Equation (26), the selected value of σ has an effect on the computation time and the solution accuracy. The larger σ is, the smoother $F_{w\sigma }\left(T_{\hat{\theta }}^{l_{2}}\right)$ will be. Larger σ can result in less local maxima and makes it more easy to maximize $F_{w\sigma }\left(T_{\hat{\theta }}^{l_{2}}\right)$. In this case, the computation process can be speeded up. However, large σ causes the deviation of the approximation in Equation (25), and it will further decrease the precision of the signal reconstruction. To guarantee both of the speed and the accuracy, a decreasing sequence for σ, i.e., $\left[\sigma_{1},\sigma_{2},\ldots ,\sigma_{K}\right]$ with $\sigma_{1}\geq \sigma_{2}\geq \ldots \geq \sigma_{K}$, which is known as the graduated non-convexity (GNC) approach [18,22], can be exploited. Even though large σ is desired to avoid a lot of local maxima and improve the speed, the value of $\sigma_{1}$ in the decreasing σ sequence may be set as one to four times of the maximum absolute value of $U_{0}^{\hat{\theta }}$. This is because in $f_{w\sigma }\left[T_{\hat{\theta }}\left(i,:\right)\right]$, when $\sigma >4\max\{|U_{0}^{\hat{\theta }}|\}$, σ acts virtually like infinity for all elements with $e^{-\frac{1}{2P\sigma^{2}}\sum_{p=1}^{P}\left[|T_{\hat{\theta }}\left(i,p\right){|}^{2}\right]}>0.96\approx 1$. Then, the other values in the σ sequence can be chosen as $\sigma_{k+1}=\alpha \sigma_{k}$ for k=1,2,…,K−1. To make the steepest ascent algorithm be not far from the actual maximum, and be less likely to get trapped into local maxima, σ is required to change slowly. This guarantees that the GNC approach can work. In general, the decreasing factor α satisfies 0.5≤α<1. $\sigma_{K}$ should be a small value determined by the desired accuracy, and a parameter $\sigma_{off}$ can be used to set the lower limit of the decreasing σ sequence.

For each $\sigma_{k}$ in the decreasing σ sequence, graduated non-convexity based *Q* iterations are carried out to maximize $F_{w\sigma }\left(T_{\hat{\theta }}^{l_{2}}\right)$. In each iteration, $T_{\hat{\theta }}$ is first updated as the following form:

(28) $$T_{\hat{\theta }}\leftarrow T_{\hat{\theta }}+\tilde{\mu }\nabla F_{w\sigma }\left(T_{\hat{\theta }}^{l_{2}}\right)=T_{\hat{\theta }}-\mu \Delta T_{\hat{\theta }},$$

where $\tilde{\mu }=\mu \sigma^{2}$ is a step-size parameter, and μ should be μ≥1. In addition, $\nabla F_{w\sigma }\left(T_{\hat{\theta }}^{l_{2}}\right)$ is the gradient of $F_{w\sigma }\left(T_{\hat{\theta }}^{l_{2}}\right)$ corresponding to $T_{\hat{\theta }}$. In the following, $\Delta T_{\hat{\theta }}$ will be derived in detail. Let the detailed expression of $T_{\hat{\theta }}$ be

(29) $$T_{\hat{\theta }}={\left[t_{1}^{T},t_{2}^{T},\ldots ,t_{L}^{T}\right]}^{T},$$

where $t_{i}\in C^{1\times P}$ is the *i*th row of $T_{\hat{\theta }}$. According to Equation (28), $t_{i}$ can be written as

(30) $$t_{i}\leftarrow t_{i}+\tilde{\mu }\nabla_{t_{i}}F_{w\sigma }\left(T_{\hat{\theta }}^{l_{2}}\right)=t_{i}-\mu \Delta t_{i},$$

where $\nabla_{t_{i}}F_{w\sigma }\left(T_{\hat{\theta }}^{l_{2}}\right)$ stands for the 1×P dimensional gradient vector of $F_{w\sigma }\left(T_{\hat{\theta }}^{l_{2}}\right)$ in regard to $t_{i}=\left[T_{\hat{\theta }}\left(i,1\right),T_{\hat{\theta }}\left(i,2\right),\ldots ,T_{\hat{\theta }}\left(i,P\right)\right]$. The *p*th element of $\nabla_{t_{i}}F_{w\sigma }\left(T_{\hat{\theta }}^{l_{2}}\right)$ is detailedly expressed as

(31) $$\begin{array}{cc} \nabla_{t_{i}}F_{w\sigma }\left(T_{\hat{\theta }}^{l_{2}}\right)\left(p\right) & =\partial F_{w\sigma }\left(T_{\hat{\theta }}^{l_{2}}\right)/\partial t_{ip}\\ & =\partial \sum_{i=1}^{L}e^{-\frac{r_{wi}}{2P\sigma^{2}}\sum_{p=1}^{P}\left[|T_{\hat{\theta }}\left(i,p\right){|}^{2}\right]}/\partial T_{\hat{\theta }}\left(i,p\right), \end{array}$$

where $t_{ip}=\tilde{a}+\tilde{b}j$ is the *p*th parameter of $t_{i}$ with the real component $\tilde{a}$ and imaginary component $\tilde{b}$. $\partial F_{w\sigma }\left(T_{\hat{\theta }}^{l_{2}}\right)/\partial t_{ip}=\left[\partial F_{w\sigma }\left(T_{\hat{\theta }}^{l_{2}}\right)/\partial \tilde{a}\right]+\left[\partial F_{w\sigma }\left(T_{\hat{\theta }}^{l_{2}}\right)/\partial \tilde{b}\right]j$ denotes the 1st-order derivative of $F_{w\sigma }\left(T_{\hat{\theta }}^{l_{2}}\right)$. As a result, $\nabla_{t_{i}}F_{w\sigma }\left(T_{\hat{\theta }}^{l_{2}}\right)\left(p\right)$ is derived as follows:

(32) $$\begin{array}{cc} \nabla_{t_{i}}F_{w\sigma }\left(T_{\hat{\theta }}^{l_{2}}\right)\left(p\right) & =e^{-\frac{r_{wi}}{2P\sigma^{2}}\sum_{p=1}^{P}\left[|T_{\hat{\theta }}\left(i,p\right){|}^{2}\right]}\partial \left\{-\frac{r_{wi}}{2P\sigma^{2}}\sum_{p=1}^{P}\left[|T_{\hat{\theta }}\left(i,p\right){|}^{2}\right]\right\}/\partial T_{\hat{\theta }}\left(i,p\right)\\ & =-\frac{r_{wi}T_{\hat{\theta }}\left(i,p\right)}{P\sigma^{2}}\exp\left\{-\frac{r_{wi}}{2P\sigma^{2}}\sum_{p=1}^{P}\left[|T_{\hat{\theta }}\left(i,p\right){|}^{2}\right]\right\}. \end{array}$$

Combining Equation (30) with Equation (32), the *p*th element of $\Delta t_{i}$ can be obtained, that is,

(33) $$\begin{array}{cc} \Delta t_{ip} & =-\sigma^{2}\nabla_{t_{i}}F_{w\sigma }\left(T_{\hat{\theta }}^{l_{2}}\right)\left(p\right)\\ & =\frac{r_{wi}T_{\hat{\theta }}\left(i,p\right)}{P}\exp\left\{-\frac{r_{wi}}{2P\sigma^{2}}\sum_{p=1}^{P}\left[|T_{\hat{\theta }}\left(i,p\right){|}^{2}\right]\right\}. \end{array}$$

With $\Delta t_{ip}$, the 1×P dimensional vector $\Delta t_{i}$ is written as

(34) $$\begin{array}{cc} \Delta t_{i} & =-\sigma^{2}\left[\partial F_{w\sigma }\left(T_{\hat{\theta }}^{l_{2}}\right)/\partial T_{\hat{\theta }}\left(i,1\right),\partial F_{w\sigma }\left(T_{\hat{\theta }}^{l_{2}}\right)/\partial T_{\hat{\theta }}\left(i,2\right),\ldots ,\partial F_{w\sigma }\left(T_{\hat{\theta }}^{l_{2}}\right)/\partial T_{\hat{\theta }}\left(i,P\right)\right]\\ & =[\Delta t_{i1},\Delta t_{i2},\ldots ,\Delta t_{iP}] \end{array}$$

for i=1,2,…,L. As a result, based on Equations (33) and (34), the sparse matrix to be recovered in each iteration is first updated as

(35) $$T_{\hat{\theta }}\leftarrow T_{\hat{\theta }}-\mu \Delta T_{\hat{\theta }}\in C^{L\times P},$$

with

(36) $$\Delta T_{\hat{\theta }}={\left[\Delta t_{1}^{T},\Delta t_{2}^{T},\ldots ,\Delta t_{P}^{T}\right]}^{T},$$

where $\Delta T_{\hat{\theta }}\in C^{L\times P}$. Then, this iteration projects $T_{\hat{\theta }}$ back to the feasible set $T_{\hat{\theta }}=\left\{T_{\hat{\theta }}|Y_{s}=FB_{\hat{\theta }}T_{\hat{\theta }}\right\},$ which satisfies Equation (20), that is,

(37) $$T_{\hat{\theta }}\leftarrow T_{\hat{\theta }}-{\left(FB_{\hat{\theta }}\right)}^{+}\left[\left(FB_{\hat{\theta }}\right)T_{\hat{\theta }}-Y_{s}\right].$$

Based on Equations (27)–(37), after *Q* iterations for $\sigma =\sigma_{k}$, the MMV based sparse signal reconstruction in Equation (26) starts the maximization process of the joint smoothed function $F_{w\sigma }\left(T_{\hat{\theta }}^{l_{2}}\right)$ for $\sigma =\sigma_{k+1}$. Let $T_{f}^{\hat{\theta }}$ be the output of $T_{\hat{\theta }}$ by maximizing $F_{w\sigma }\left(T_{\hat{\theta }}^{l_{2}}\right)$ for $\sigma =\sigma_{K}$. Consequently, the final sparse matrix is recovered as $T_{f}^{\hat{\theta }}$. In order to evaluate the reconstruction results of each row in $T_{f}^{\hat{\theta }}$, and then achieve the DOA estimation, let

(38) $${\bar{\ell }}_{p}={\left(T_{f}^{\hat{\theta }}\right)}^{l_{2}},$$

with $\left[{\left(T_{f}^{\hat{\theta }}\right)}^{l_{2}}\right]\left(i\right)=∥T_{f}^{\hat{\theta }}\left(i,:\right){∥}_{2}$. Therefore, by searching the spectrum of ${\bar{\ell }}_{p}$, the target DOAs are obtained. The summary of the proposed joint smoothed $l_{0}$-norm algorithm is listed below:

Step 1: Compute the fourth-order cumulants based matrix $Y_{s}$ from Equations (11), (16) and (19).
Step 2: Design the joint smoothed function $f_{w\sigma }\left[T_{\hat{\theta }}\left(i,:\right)\right]$ tailored for the MMV case as Equation (23).
Step 3: Construct the joint smoothed $l_{0}$-norm sparse representation framework in Equation (26).
Step 4: Execute the fast MMV-based sparse signal reconstruction with Equations (27)–(37).
Step 5: Attain the DOA estimation based on Equation (38).

## 4. Related Remarks

**Remark** **1.** *With the joint smoothed function $F_{w\sigma }\left(T_{\hat{\theta }}^{l_{2}}\right)$, the proposed joint smoothed $l_{0}$-norm minimization based sparse signal reconstruction algorithm is a new fast sparse DOA estimation algorithm, which is tailored for the MMV problem. By designing a specific joint continuous function that exploits all data information in the data matrix $Y_{s}$, and then, deriving its steepest ascent process to achieve the maximization, the sparse solutions can be obtained. In the extended and improved smoothed $l_{0}$-norm algorithms, the proposed joint continuous function $f_{\sigma }\left[T_{\hat{\theta }}\left(i,:\right)\right]$ in Equation (21) is applicable for all MMV cases with the form $Y_{s}={\hat{B}}_{\hat{\theta }}T_{\hat{\theta }}$, where ${\hat{B}}_{\hat{\theta }}$ is the complete dictionary and $T_{\hat{\theta }}$ is the L×P dimensional sparse matrix that needs to be reconstructed. Under this circumstance, gradient-based maximizing $\sum_{i=1}^{L}f_{\sigma }\left[T_{\hat{\theta }}\left(i,:\right)\right]$ is similar to the derivation of Equations (27)–(37), and its $\Delta t_{ip}$ corresponding to $\sum_{i=1}^{L}f_{\sigma }\left[T_{\hat{\theta }}\left(i,:\right)\right]$ is concluded as*

(39) $$\begin{array}{c} \Delta t_{ip}=\frac{T_{\hat{\theta }}\left(i,p\right)}{P}\exp\left\{-\frac{1}{2P\sigma^{2}}\sum_{p=1}^{P}\left[|T_{\hat{\theta }}\left(i,p\right){|}^{2}\right]\right\} \end{array}$$

*for i=1,2,…,L. With $\Delta t_{ip}$ in Equation (39), the proposed joint smoothed $l_{0}$-norm algorithm regarding the joint continuous function $f_{\sigma }\left[T_{\hat{\theta }}\left(i,:\right)\right]$ can be applied to other fast sparse signal reconstruction problems that involve multiple measurement vectors.*

**Remark** **2.** The proposed method has low computational complexity because its sparse signal recovery is a graduated non-convexity procedure, rather than the convex optimization problem involved in the $l_{1}$-norm minimization. When recovering the sparse signal matrix $T_{\hat{\theta }}$, the main computational burden is caused by the QK iterations, which require about O[PLQK+(M+N−1)PLQK], where L is the number of the sample grid cells in the complete dictionary $B_{\hat{\theta }}$. For the SMV based reweighted smoothed $l_{0}$-norm algorithm in [22], recovering the sparse signal vector requires about $O[{\left(M+N-1\right)}^{2}LQK]$. However, $l_{1}$-norm minimization based DOA estimation methods, such as $l_{1}$-SVD and RV $l_{1}$-SVD, require $O[{\left(PL\right)}^{3}]$ and $O[\frac{1}{4}{\left(PL\right)}^{3}]$, respectively. In addition, the RV $l_{1}$-SRACV needs $O[\frac{1}{4}L^{3}]$. As L is much larger than P, M+N−1, Q and K, the computation speed of the proposed joint smoothed $l_{0}$-norm algorithm is much faster than the $l_{1}$-norm minimization based methods.

**Remark** **3.** The proposed method can deal with both of the white and colored Gaussian noises, thereby making it desirable for use in practical applications. This is because, in the proposed joint smoothed $l_{0}$-norm algorithm, high-order cumulants are utilized to process the reduced-dimensional data x(t). Note that, for the dimension reduction in Equation (11), the reduced-dimensional matrix R satisfies $RR^{H}=I_{M+N-1}$. Hence, if the noise vector $\tilde{n}\left(t\right)$ is white Gaussian with covariance matrix $\rho^{2}I_{MN}$, it remains white Gaussian with covariance matrix $R\rho^{2}I_{MN}R^{H}=\rho^{2}I_{M+N-1}$ after the transformation [2,25]. On the other hand, if the noise vector n(t) is colored Gaussian with covariance matrix $V_{a}$, its covariance matrix turns into $RV_{a}R^{H}$ after the dimensional reduction. However, in this case, $\tilde{n}\left(t\right)$ is still Gaussian according to the invariance speciality of linear transformation in asymptotic normal distribution. Therefore, even though the dimensional reduction makes the change of the noise happen, the high-order cumulant process in Equation (16) can eliminate it.

## 5. Simulation Results

In this part, some simulations are implemented to evaluate the performance and the computation speed of the proposed method. The simulation results of the proposed method are compared with those of the $l_{1}$-SVD [7], RV $l_{1}$-SVD [16], RV $l_{1}$-SRACV [14] and reweighted smoothed $l_{0}$-norm (RSL0) [22] algorithms. In all of the simulations, a narrowband monostatic MIMO radar system with ULAs is considered. The antennas of the transmit array and the receive array are both half-wavelength spaced, and their numbers are M=6 and N=6, respectively. The transmitted signals are assumed to be BPSK modulated [27,30], moreover, they are mutually orthogonal and different from each other. The noise is white Gaussian with the covariance matrix $R=I_{MN}$, or colored Gaussian with the elements in the covariance matrix being $R\left(k_{1},k_{2}\right)=0.75^{|k_{1}-k_{2}|}e^{j\pi (k_{1}-k_{2})/2}$. By means of the minimum description length (MDL) principle or the Akaike information criterion (AIC) principle [31], the prior knowledge of the target number is assumed to be known as *P*.

When constructing the complete dictionary, the discrete sample grid is set from $-90^{\circ }$ to $90^{\circ }$ with the uniform interval distance $\Delta \hat{\theta }=0.05^{\circ }$ for the proposed algorithm, as well as the $l_{1}$-SVD, RV $l_{1}$-SVD, RV $l_{1}$-SRACV and RSL0 algorithms. The parameters $\sigma_{1}$, μ and $\sigma_{off}$ are fixedly set at $\sigma_{1}=4\max\left\{|U_{0}^{\hat{\theta }}|\right\}$, μ=2.5 and $\sigma_{off}=0.0007$ for the proposed algorithm and the RSL0 algorithm. The effects of the other parameters, i.e., the decreasing factor α and the iteration number *Q*, will be evaluated and given in detail in the following experiments. The definitions for the signal-to-noise ratio (SNR) and the root mean square error (RMSE) are shown, respectively, as follows:

(40) $$\begin{array}{cc} \mathrm{SNR} & =10\log_{10}{\left(||AS|\right|}_{F}^{2}/{\left||N|\right|}_{F}^{2}),\\ \mathrm{RMSE} & =\sqrt{\frac{1}{500P}\sum_{i=1}^{500}\sum_{p=1}^{P}{\left({\hat{\theta }}_{p,i}-\theta_{p}\right)}^{2}}, \end{array}$$

where ${\hat{\theta }}_{p,i}$ is the angle estimation of the *p*th target DOA $\theta_{p}$ for the *i*th Monte Carlo trial.

**Figure 2:**
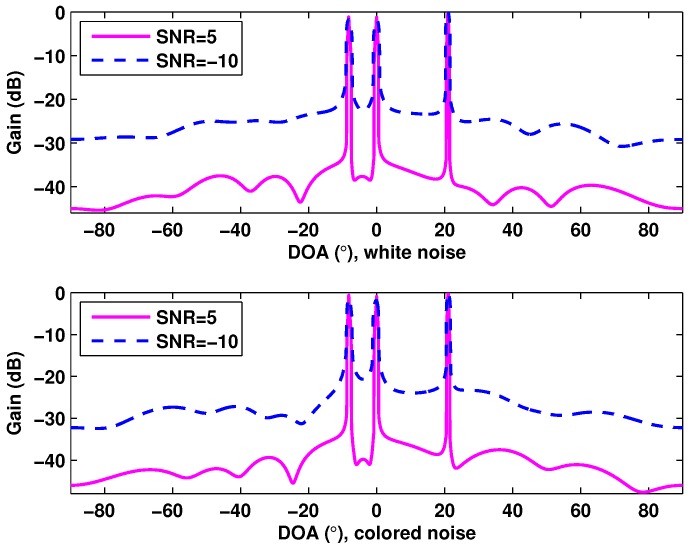
Spatial spectrum of the proposed method for white noise and colored noise.

Figure 2 depicts the spatial spectrum of the proposed method for white noise and colored noise, where SNR = 5 dB and SNR = −10 dB are considered. There are three targets located at $\theta_{1}=-8.2^{\circ }$, $\theta_{2}=0^{\circ }$ and $\theta_{3}=21^{\circ }$. The spatial spectrum is plotted by computing $10\log_{10}\left[\right|{\bar{\ell }}_{p}\left|/\max(\right|{\bar{\ell }}_{p}\left|)\right]$ with ${\bar{\ell }}_{p}$ being the final sparse solution. Figure 2 shows that the proposed joint smoothed $l_{0}$-norm minimization algorithm is effective for the MMV case of DOA estimation in monostatic MIMO radar. Moreover, it is suitable for the practical radar systems with colored Gaussian noise.

**Figure 3:**
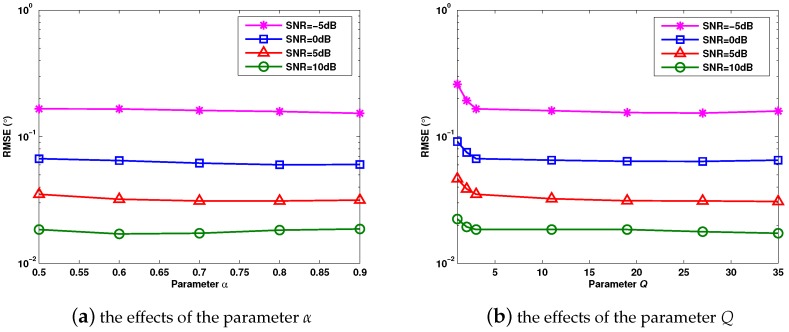
RMSE (root mean square error) versus different values of the parameters α and *Q*.

Figure 3a,b evaluates the effects of the parameters α and *Q* on the DOA estimation performance of the proposed method, with Q=3 and α=0.5, respectively. Moreover, J=1000. Three uncorrelated targets with the DOAs $\theta_{1}=-8.2^{\circ }$, $\theta_{2}=0^{\circ }$ and $\theta_{3}=21^{\circ }$ are considered. In Figure 3, it can be verified that the proposed method keeps the same angle estimation performance when the parameters α and *Q* vary from α=0.5 to α=0.9 and Q=3 to Q=35. In Figure 3b, the RMSE decreases with the increase of *Q* when Q≤3, and it reaches an asymptotic value at Q=3. For the iterations of the steepest ascent algorithm, we just need a region near the maximum value of $F_{w\sigma }\left(T_{\hat{\theta }}^{l_{2}}\right)$; thus, a fixed small positive integer Q=3 is applicable.

With respect to Figure 3, the effects of the parameters α and *Q* on the average computation time of the proposed method are shown in Table 1 and Table 2. The simulation conditions keep the same with those of Figure 3. As the implementing processes of the analyzed methods are not affected by the type of the noise, the computation time is the same for the white and the colored Gaussian noises. It can be observed that the computation speed becomes slower when α and *Q* increase. However, larger α and *Q* are not able to improve the estimation performance of the proposed method, as shown in Figure 3a,b. As a result, small values of α and *Q* are enough to guarantee the estimation accuracy and the computation speed. In the following experiments, the parameters α and *Q* are set at α=0.5 and Q=3.

**Figure 4:**
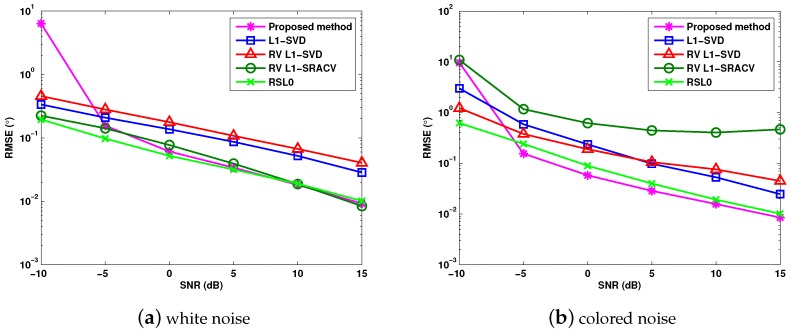
RMSE versus SNR (signal-to-noise ratio) for different sparse DOA (direction-of-arrival) estimation algorithms.

Figure 4a,b verify the RMSE of DOA estimation versus SNR for white noise and colored noise, where J=1000. Consider three targets whose DOAs are $\theta_{1}=-8.2^{\circ }$, $\theta_{2}=0^{\circ }$ and $\theta_{3}=21^{\circ }$. According to Figure 4a,b, we can conclude that the RSL0 algorithm achieves the best DOA estimation for white noise. For colored noise, the proposed algorithm and the RSL0 algorithm provide better angle estimation performance than the $l_{1}$-SVD, RV $l_{1}$-SVD and RV $l_{1}$-SRACV methods.

The processing time of signal reconstruction is compared in Table 3, where J=1000 and SNR = 0 dB. Different antenna numbers and target numbers are considered. For p=2, the DOAs are $\theta_{2}=0^{\circ }$ and $\theta_{3}=21^{\circ }$. For p=3, they are $\theta_{1}=-8.2^{\circ }$, $\theta_{2}=0^{\circ }$ and $\theta_{3}=21^{\circ }$. Table 3 verifies that, compared with the $l_{1}$-norm minimization based methods, the proposed algorithm has much lower computational complexity and is about two orders of magnitude faster.

**Figure 5:**
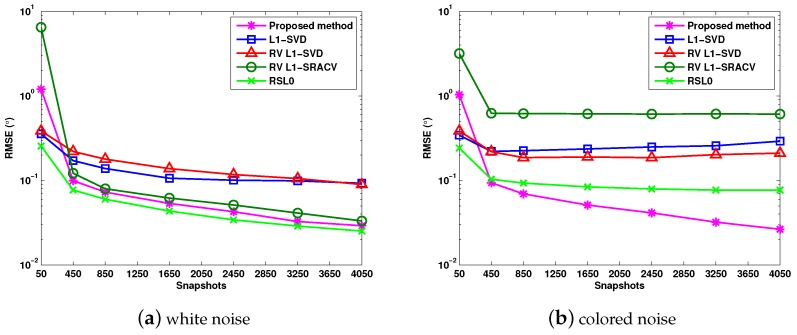
RMSE versus snapshots with SNR = 0 dB.

Figure 5a,b illustrates the RMSE versus snapshots for Gaussian white noise and colored noise, where SNR = 0 dB, and the DOAs of three targets are $\theta_{1}=-8.2^{\circ }$, $\theta_{2}=0^{\circ }$ and $\theta_{3}=21^{\circ }$. It can be observed that, for white noise, the RSL0 algorithm has the minimum RMSE. For colored noise, the proposed algorithm provides the best angle estimation performance when the snapshots vary from J=450 to J=4050, whereas, in this case, the RV $l_{1}$-SRACV method performs worse than the other algorithms.

**Figure 6:**
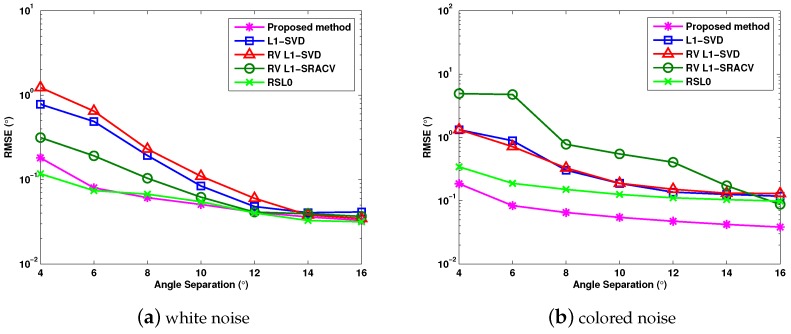
RMSE versus angle separation with SNR = 0 dB.

Figure 6a,b verifies the DOA estimation performance of different sparse methods versus angle separation for Gaussian white noise and colored noise, where J=1000, and the DOAs of two targets are considered as $\theta_{1}=0^{\circ }$ and $\theta_{2}=\theta_{1}+\tilde{\theta }$ with $\tilde{\theta }$ varying from $\tilde{\theta }=4^{\circ }$ to $\tilde{\theta }=16^{\circ }$. For the white noise in Figure 6a, the RSL0 algorithm in [22] performs the best, and the proposed algorithm provides better estimation performance than the $l_{1}$-SVD, RV $l_{1}$-SVD and RV $l_{1}$-SRACV methods. For the colored noise in Figure 6b, the performance of the proposed algorithm stands out among the analyzed methods, while the performance of the RV $l_{1}$-SRACV is severely degraded.

**Figure 7:**
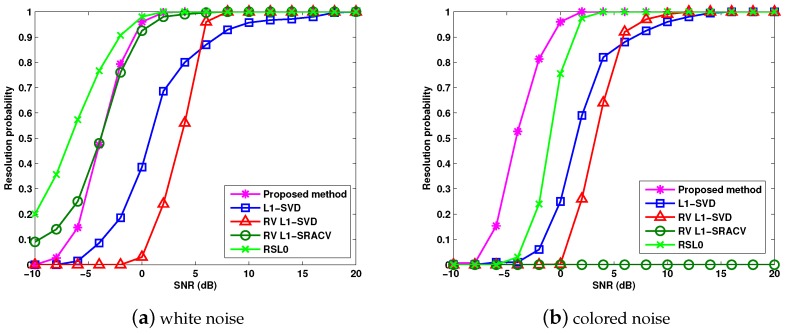
RMSE versus target resolution probability.

Figure 7a,b verifies the relationships between the target resolution probability and SNR for different sparse methods, where J=1000, and three targets $\theta_{1}=-8.2^{\circ }$, $\theta_{2}=0^{\circ }$ and $\theta_{3}=21^{\circ }$ are considered. The *i*th Monte Carlo trial is regarded as a successful one if the estimation results ${\bar{\theta }}_{1}$, ${\bar{\theta }}_{2}$ and ${\bar{\theta }}_{3}$ satisfy $\max\{|{\bar{\theta }}_{1}-\theta_{1}\left|,\right|{\bar{\theta }}_{2}-\theta_{2}\left|,\right|{\bar{\theta }}_{3}-\theta_{3}\left|\right\}\leq 0.1^{\circ }$. Figure 7a,b verifies that the RSL0 algorithm performs the best for the white noise, and the proposed algorithm provides the highest target resolution probability for the colored noise. Colored noise causes the serious performance degradation for the analyzed methods except for the proposed algorithm.

## 6. Conclusions

Direction-of-arrival estimation is usually confronted with the MMV case. In this paper, for DOA estimation in MIMO radar, we have proposed a joint smoothed $l_{0}$-norm algorithm tailored for the multiple measurement vector case. By designing the new joint smoothed function and its gradient-based sparse signal reconstruction, the proposed method is a fast sparse DOA estimation algorithm that can solve the MMV problem. In addition, it is applicable to the colored Gaussian noise. The extended applications and the computational complexity of the proposed method are analyzed. The experimental results have verified that the proposed algorithm is about two orders of magnitude faster than the $l_{1}$-norm minimization based methods, such as $l_{1}$-SVD, RV $l_{1}$-SVD and RV $l_{1}$-SRACV, and performs well for both white and colored Gaussian noises.

## Figures and Tables

**Table 1:** Average computation time for different values of the parameter α.

α	0.5	0.6	0.7	0.8	0.9
**Average Computation Time (s)**	0.0401	0.0498	0.0752	0.1137	0.2511

**Table 2:** Average computation time for different values of the parameter *Q*.

Q	3	11	19	27	35
**Average Computation Time (s)**	0.0399	0.1382	0.2576	0.3696	0.4738

**Table 3:** Average computation time of the signal reconstruction for different algorithms.

(M,N,P)	Average Computation Time (s)				
	Proposed Method	$l_{1}$-SVD	RV $l_{1}$-SVD	RV $l_{1}$-SRACV	RSL0
**(4,4,2)**	0.0269	2.1067	1.5499	1.3213	0.0135
**(4,4,3)**	0.0323	2.5139	2.0591	1.3368	0.0139
**(5,5,2)**	0.0273	2.4984	2.1071	2.1277	0.0252
**(5,5,3)**	0.0373	3.1170	2.7076	2.3842	0.0254
**(6,6,2)**	0.0328	2.6323	2.2911	3.2908	0.0438
**(6,6,3)**	0.0395	3.5439	3.1187	4.7397	0.0441

SVD (singular value decomposition), RV (real-valued), SRACV (sparse representation array covariance vectors), RSL0 (reweighted smoothed $l_{0}$-norm).

## Abbreviations

The following abbreviations are used in this paper:

MIMO	Multiple-Input Multiple-Output
DOA	Direction-of-Arrival
FOC	Fourth-Order Cumulants
MMV	Multiple Measurement Vector
SMV	Single Measurement Vector
SVD	Singular Value Decomposition
ACV	Array Covariance Vectors
SR	Sparse Representation
RV	Real-Valued
